# How Does Leader Empowering Behavior Promote Employee Knowledge Sharing? The Perspective of Self-Determination Theory

**DOI:** 10.3389/fpsyg.2021.701225

**Published:** 2021-08-31

**Authors:** Shuting Xiang, Yuan Zhang, Nan Ning, Shan Wu, Weiru Chen

**Affiliations:** School of International Business, Southwestern University of Finance and Economics, Chengdu, China

**Keywords:** leader empowering behavior, knowledge sharing, self-determination theory, proactivity, moderated mediation

## Abstract

Although scholars have recognized the important role of leader empowering behavior in promoting employee knowledge sharing, investigations on the potential underlying mechanism are still limited. To enrich studies revealing the possible underlying paths, drawing on self-determination theory, this paper proposes a moderated mediation model. We propose that employee self-determination plays a mediating role and employee proactivity moderates the mediating effect. We test our hypotheses based on data collected from 230 employees across a three-wave study. The empirical results demonstrate that leader empowering behavior promotes employee knowledge sharing by enhancing employee self-determination. Employees’ proactive personality moderates the indirect effect such that the indirect effect is stronger when employees have high levels of proactive personality. This paper thus contributes to the related literature and reveals practical implications.

## Introduction

To cope with fierce competition and dynamic environmental change, the importance of knowledge management has been emphasized, indicating that knowledge is an important strategic resource of organizations (Spender and Grant, 1996; Davenport and Prusak, 1998; Lei et al., 2021). Since knowledge sharing is the key to creating and utilizing knowledge (Nahapiet and Ghoshal, 1998; Wasko and Faraj, 2000; Le and Son, 2020), an increasing number of researchers have emphasized knowledge sharing in the field of knowledge management. Previous research has shown that employees’ perceived leader support can promote employee knowledge sharing (Cabrera et al., 2006; Carmeli et al., 2013; Hao et al., 2019), and different leadership styles have different impacts on employee knowledge sharing. For example, transformational leadership promotes knowledge sharing among employees (Kelloway and Barling, 2000; Liu and Li, 2018; Le and Son, 2020). Ethical leadership is positively related to employee knowledge sharing through the mediating effects of controlled motivation and moral identity (Bavik et al., 2018). Humble leadership promotes employee knowledge sharing through psychological safety (Wang et al., 2018). By contrast, transactional leadership is less effective in promoting knowledge sharing (Bryant, 2003). Abusive supervision negatively affects employee knowledge sharing through emotional exhaustion (Lee et al., 2017).

Currently, enterprises are beginning to change their organizational structure, reduce the organizational hierarchy, and transform from the traditional management structure to empowered teamwork (Arnold et al., 2000). Accordingly, the role of leaders has transformed into supporting working groups, encouraging employee self-management, and promoting empowerment, which can be viewed as leader empowering behavior. Leader empowering behavior refers to leaders’ top-down assignment of responsibilities to subordinates, granting subordinates more decision-making power to complete their tasks (Leach et al., 2003; Cheong et al., 2016; Smallfield et al., 2020). Research has demonstrated that leader empowering behavior can promote employee knowledge sharing (Srivastava et al., 2006; Xue et al., 2011; Chuang et al., 2016). Researchers have also explored the underlying mechanism of the influence of leader empowering behavior on employee knowledge sharing. For example, Wu and Lee (2017) applied social exchange theory and revealed that psychological capital played a mediating role in the relationship between leader empowering behavior and employee knowledge sharing. Usman et al. (2021) found that empowering leadership affected employee knowledge sharing through psychological empowerment.

These studies reveal how leader empowering behavior promotes employee knowledge sharing. However, investigations on the possible paths of influence are still limited. To deepen the study of knowledge-sharing motivation, Gagné (2009) proposed a model based on the theory of planned behavior (Ajzen, 1991) and self-determination theory (SDT; Deci and Ryan, 1985, 2000), indicating the important role of autonomous motivation and need satisfaction in explaining employee knowledge-sharing behavior. Responding to the call exploring what influences knowledge sharing from the perspective of self-determination (Gagné, 2009), Gagné et al. (2019) first tested the model empirically and suggested that autonomous motivation was positively related to knowledge sharing.

According to SDT, individuals’ autonomous motivation is influenced by the degree to which their basic psychological needs for autonomy, competence, and relatedness are satisfied (Ryan and Deci, 2000). The satisfaction of these three basic psychological needs plays a role in the development toward self-determination (Gagné and Deci, 2005). When people become self-determinate, they are more likely to share knowledge (Gagné, 2009; Coun et al., 2019). Furthermore, Gagné’s (2009) model showed that several important human resource management practices, including motivating job design, motivating managerial styles, and training, enhance need satisfaction. These motivational characteristics fit well with leader empowering behavior, which highlights the significance of the work, fosters participation in decision making, expresses confidence in high performance, and provides autonomy (Ahearne et al., 2005). We believe that empowering behavior may promote employees’ basic psychological needs satisfaction, facilitate their self-determination, and in turn promote knowledge sharing. However, to the best of our knowledge, no studies have investigated the mediating role of self-determination in the relationship between leader empowering behavior and employee knowledge sharing. Therefore, in this paper, we aim to investigate whether leader empowering behavior enhances employee self-determination and further promotes knowledge sharing.

**Figure 1:**
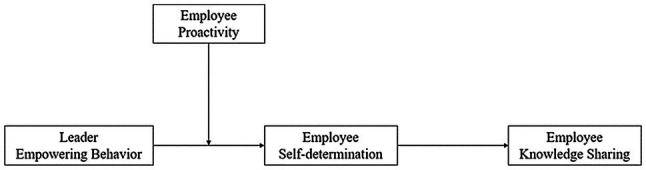
Theoretical Model.

On this basis, we also explore the boundary conditions. Gagné and Deci (2005) pointed out that when drawing on SDT to study individual motivation, the interactive effect of the social environment and individual differences (i.e., individual personality) on motivation should be considered. In this paper, we aim to explore the moderating effect of employee proactive personality on the relationship between leader empowering behavior and employee knowledge sharing. In recent years, scholars have begun to explore the moderating effect of proactive personality. For instance, in the field of entrepreneurial behavior, proactive personality can moderate the positive association between entrepreneurial intention and entrepreneurial behavior (Neneh, 2019; Li et al., 2020). The study conducted by Jafri et al. (2016) proved that proactive personality can strengthen the positive relationship between emotional intelligence and employee creativity. Individuals with a proactive personality commonly have high levels of competence, initiative, engagement, and other positive characteristics (Campbell, 2000; Newman et al., 2017). We believe that when facing leader empowering behavior, employees with such competences and initiative find their needs satisfied and thus promote their self-determination and knowledge sharing. Therefore, we also investigate whether employee proactive personality moderates the relationship between leader empowering behavior and employee knowledge sharing through the mediating role of self-determination. The theoretical model is shown in Figure 1.

Our paper offers three key contributions to the literature. First, it extends the literature on leader empowering behavior by examining how it promotes employee self-determination, which is lacking in previous studies (Coun et al., 2019). It also expands Gagné’s (2009) model of knowledge-sharing motivation by studying the role of leader behavior in facilitating employee knowledge-sharing motivation. Second, we contribute to the literature on the underlying mechanism of how leader empowering behavior influences employee knowledge sharing. Previous studies neglect the role of basic psychological needs satisfaction in this relationship (i.e., Wu and Lee, 2017; Usman et al., 2021). By investigating the mediating role of self-determination based on 230 employees from a Chinese R&D company, we fill this gap and enrich the relevant research. Third, drawing on SDT and exploring the interactive effect of leader empowering behavior and employee proactive personality on employees’ self-determination and knowledge sharing, we also expand Gagné’s (2009) model by clarifying the boundary conditions under which employees may perceive high levels of need satisfaction and engage in knowledge sharing.

## Theoretical Review and Research Hypotheses

### Leadership Empowering Behavior and Employee Knowledge Sharing

There exists no uniform definition for knowledge sharing. Bartol and Srivastava (2002) defined knowledge sharing as information, ideas, opinions, and expertise that individuals share with others. Some scholars define knowledge sharing as providing information, knowing how to help others and cooperating with others to solve problems, propose new ideas, and implement policies and procedures (Pulakos et al., 2003; Cummings, 2004). From the perspective of process, Hendriks (1999) believes that knowledge sharing includes not only the externalization process of knowledge owners, but also the internalization process of knowledge requesters. The definition proposed by Cummings (2004) is similar. Our study focuses on the “providing process” of knowledge sharing and defines knowledge sharing as the behavior of individuals providing work-related information, expertise, technology, experience, ideas, and methods to others. Individual knowledge sharing has been shown to improve personal decision-making ability (Voelpel et al., 2005), reduce organizational production costs, shorten project completion time (Hansen, 2002), promote organizational innovation (Lin, 2007), and improve organizational performance (Collins and Smith, 2006). Therefore, how to motivate individuals to better share knowledge is crucial to organizations.

Previous studies have shown that leader behavior has an important impact on employee knowledge sharing. For example, employees’ knowledge sharing is supported by perceived support from leaders and colleagues (Connelly and Kelloway, 2003; Cabrera et al., 2006), leaders’ commitment to knowledge sharing (Martiny, 1998), and transformational leadership (Kelloway and Barling, 2000). In this study, we aim to explore the possible impact of leader empowering behavior on employee knowledge sharing. Leader empowering behavior refers to leaders’ top-down assignment of responsibilities, which allows subordinates more decision-making power to complete their tasks (Leach et al., 2003). According to Ahearne et al. (2005) conceptualization, leader empowering behavior contains four dimensions: enhancing the meaningfulness of work, fostering participation in decision making, expressing confidence in high performance, and providing autonomy from bureaucracy. We suggest that leader empowering behavior has a positive impact on employee knowledge-sharing behavior for the following reasons:

First, leader empowering behavior enhances the meaningfulness of work and may thus lead employees to feel that knowledge sharing is valuable and to be more inclined to share knowledge. Since leaders help employees understand how important their contributions are to organizational effectiveness (Zhang and Bartol, 2010), employees may recognize the importance and meaningfulness of knowledge sharing and thus become more likely to share knowledge (Welschen et al., 2012). Second, by fostering employee participation in decision making, leaders unify the organization’s goals and the employee’s goals. When goals are consistent, there may be a state of “swimming or sinking together.” This kind of state is conducive to mutual support, mutual trust, cooperation, and open discussion among employees, thus fostering employee knowledge sharing (Wasko and Faraj, 2000; Wang and Yen, 2012). Third, by expressing confidence in an employee’s competence, leader empowering behavior enhances employee knowledge sharing through self-efficacy. When leaders expect high performance, employees may feel that they are competent to accomplish the job. The sense of self-efficacy may facilitate their involvement in sharing knowledge with others (Cabrera et al., 2006; Lu et al., 2006; Hsu et al., 2007). Fourth, providing autonomy to employees fosters their autonomous motivation, which promotes knowledge-sharing behavior. Through empowerment, leaders commonly encourage employees to engage in independent action (Pearce and Sims, 2002; Ahearne et al., 2005). Then, employees may feel intrinsically motivated to work or recognize the meaningfulness of their work. Additionally, some prior research can support our hypothesis. Srivastava et al. (2006) proved that empowering leader can promote knowledge sharing inside teams. More recently, Tang et al. (2020) proposed that leaders’ empowerment can contribute to a shared mindset and higher predictability in their managed teams, which can encourage exchange and share of new information and perspectives. Accordingly, we hypothesize as follows:

*H1*: Leader empowering behavior is positively related to employee knowledge sharing.

### The Mediating Role of Self-Determination

According to SDT, individuals have three basic psychological needs: autonomy, competence, and relatedness (Ryan and Deci, 2000; Gagné and Deci, 2005). The need for autonomy (NFA) refers to individuals’ need for control and autonomy over their behaviors and choices regarding their own behaviors. The need for competence (NFC) refers to the need to master assignments and be recognized by others. The need for relatedness (NFR) refers to the need to be related to and recognized by others (Ryan and Deci, 2000). The higher the degree to which these needs are satisfied, the stronger the sense of self-determination individuals perceive (Gagné and Deci, 2005). We argue that leader empowering behavior promotes the satisfaction of employees’ autonomy, competence, and relatedness needs, thus enhancing employees’ sense of self-determination. The logic is as follows.

By fostering participation in decision making and providing autonomy from bureaucratic sources, leader empowering behavior enables employees to work independently, thus enhancing the satisfaction of their autonomy needs. Specifically, by involving employees in setting their own goals and encouraging employees to find solutions independently and think about learning opportunities (Pearce and Sims, 2002), leader empowering behavior enables employees to choose their own job goals, modes, and means, which enhances their sense of autonomy. Furthermore, leader empowering behavior has been shown to enhance employees’ perceived psychological empowerment (Conger and Kanungo, 1988; Thomas and Velthouse, 1990; Kirkman and Rosen, 1999). Employees can choose how to complete their own work, which is helpful to meet their autonomy needs.

By expressing confidence in high performance, leader empowering behavior enhances the satisfaction of employees’ competence needs. Specifically, when leaders suggest that employees are competent to accomplish their work, employees may feel that their abilities are recognized by the leader, which meets their competence needs. Moreover, employees may adjust their behavior based on the social cues they perceive (Salancik and Pfeffer, 1978). When leaders expect high performance, employees are more likely to improve their abilities and competence to accomplish job requirements and tasks. Thus, they feel that they are competent in their job and gain an enhanced sense of competence. In short, leader empowering behavior enhances the satisfaction of employees’ competence needs by recognizing their competence and improving their actual competence.

By fostering employee participation in decision making, leader empowering behavior provides employees with opportunities to communicate with leaders and other team members, thus enhancing the satisfaction of employees’ relatedness needs. Specifically, by encouraging employees to make decisions together as a team, leader empowering behavior fosters team cohesiveness and consistency (Tung and Chang, 2011; Hon and Chan, 2013) and trust among colleagues (Xue et al., 2011), which helps meet employees’ relatedness needs. In summary, leader empowering behavior may enhance employees’ sense of self-determination by meeting their autonomy, competence, and relatedness needs. Accordingly, we hypothesize the following:

*H2*: Leader empowering behavior is positively related to employees’ self-determination.

When employees’ autonomy, competence, and relatedness needs are satisfied, they develop a sense of self-determination, which may promote their knowledge-sharing behavior. Specifically, when employees’ autonomy needs are met, they feel more initiative in their work. In this situation, employees are not given detailed guidance, so they can determine their own job goals and must find effective ways to carry out their work independently. In this process, sharing ideas and experience with colleagues is an effective way to improve work performance. As a result, employees tend to become more willing to share knowledge with colleagues. Previous research has shown that job autonomy can promote knowledge sharing among employees (Cabrera et al., 2006).

When employees’ competence needs are met, they feel that they have the ability to share knowledge, which promotes their knowledge sharing. Individual knowledge-sharing behavior largely depends on whether individuals have the ability to share knowledge, which relates to whether they have the corresponding knowledge and experience. When employees’ competence needs are met, they feel competent for their work and have confidence in their work experience and knowledge. Previous research has shown that when individuals are confident about what they share, they are likely to share knowledge with others (Wasko and Faraj, 2005). In addition, employees’ competence needs can enhance their sense of self-efficacy, which may promote knowledge sharing (Bartol and Locke, 2000; Lu et al., 2006; Hsu et al., 2007).

When employees’ relatedness needs are met, they often identify strongly with their team or organization, which promotes their knowledge sharing. Employees whose relatedness needs are met are often recognized by others (Ryan and Deci, 2000). Therefore, they are likely to trust and identify with others in the team or organization. Previous research has shown that knowledge sharing occurs when individuals have a high level of trust in the team and organization (Hinds and Pfeffer, 2003). Trust, cooperation, and reciprocity in a team or organization also facilitate knowledge sharing directly (Constant et al., 1994; Wasko and Faraj, 2000; Reagans and McEvily, 2003). Therefore, when employees perceive high levels of self-determination, they are inclined to share knowledge. Accordingly, we hypothesize the following:

*H3*: Employees’ self-determination is positively related to their knowledge sharing.

Self-determination theory (Deci and Ryan, 1985, 1994) proposes that human beings are proactive organisms who actively internalize social cues into personal values or self-regulation, which are integrated to form personal motivation and produce corresponding behaviors. Therefore, individual motivation is an important mediating mechanism in the process by which the social context affects individual behavior. We argue that the influence of leader empowering behavior on employee knowledge sharing is mediated by employees’ self-determination. Specifically, employees’ needs for autonomy, competence, and relatedness are met when they are empowered by leaders. Then, they internalize empowerment and integrate it into their personal motivation to produce a sense of self-determination. When employees perceive high levels of self-determination, they feel more autonomy, self-efficacy, trust, and identification with others, thus promoting their knowledge sharing (Bartol and Locke, 2000; Reagans and McEvily, 2003; Cabrera et al., 2006; Hsu et al., 2007). Accordingly, we hypothesize the following:

*H4*: The relationship between leader empowering behavior and employee knowledge sharing is mediated by employees’ self-determination.

### The Moderating Role of Proactive Personality

People’s perceptions and reactions vary even when they are faced with identical stimuli. Most studies have applied the Big Five model to explain the difference in individuals’ personalities. However, Bateman and Crant (1993) found that the difference in people’s proactive behavior to change the external environment cannot be explained by the Big Five model. Therefore, they proposed a proactive personality, which describes people’s inclination to behave actively to impact the external environment and create changes. Empirical studies have stated that individuals with high proactive characteristics prefer to change their current environment, resist the limitations of contextual barriers, excel in identifying and taking advantage of opportunities, exploit their initiatives, and conduct actions to reach their goals or await new opportunities (Crant, 2000). We posit that a proactive personality positively moderates the positive relationship between leaders’ empowering behavior and employees’ self-determination such that the correlation is higher when individuals have a more proactive personality. This can be explained by the combination of the satisfaction of autonomy, competence, and relatedness stimulated by high proactivity.

As discussed above, leaders’ empowerment can boost their followers’ self-determination by satisfying their needs for autonomy, competence, and relatedness. Foremost, as proactive individuals are forward looking (Neneh, 2019) and search for opportunities (Bateman and Crant, 1993), they excel at using environmental cues to identify opportunities. In addition to providing resources, leaders’ empowerment can bring potential opportunities to their managed staff, such as the opportunity to act independently. Highly proactive individuals can efficiently grasp upcoming opportunities, insist on own objectives despite of uncertainties (Neneh, 2019), and effectively take advantage of them owing to their high degree of initiative. In turn, they can utilize the opportunities created by leader empowerment to improve their abilities or make changes that lead to better job performance and career success (Fuller and Marler, 2009). Consequently, their NFC can be satisfied.

Second, individuals with high levels of proactivity find that their NFR is better satisfied when they are empowered by leaders. Proactive employees are eager to discuss and exchange ideas with their leaders (Fuller and Marler, 2009; Li et al., 2010). When their leaders encourage them to participate in decision making, they communicate with their leaders more actively. Then, the leaders reciprocate their support and loyalty with a greater allocation of resources (Li et al., 2010). This can yield a better relationship with their leaders, as proved by Li et al. (2010), who found that proactivity is positively associated with leader-member exchange (LMX). Thus, the positive relationship between leader empowerment and the NFR can be strengthened by proactivity. Another point is that empowering leaders encourage teamwork (Pearce and Sims, 2002). Proactive employees are willing and active to communicate with and assist other members during their cooperation. In this way, they can not only enhance team effectiveness (Pearce and Sims, 2002) but also gain better connections, more respect, better reputation, more trust, and higher status during their interaction with their colleagues. Consequently, when employees are characterized by high proactivity, their relatedness needs can be met with the autonomy granted by their supervisors.

Third, proactive employees obtain a higher degree of autonomy when they are empowered by leaders. This is because proactive people seek and utilize opportunities in their environment for self-development (Bateman and Crant, 1993). When their supervisors provide them autonomy, such as participating in goal setting, working independently, and solving problems independently, proactive individuals are sensitive to these opportunities and actively take advantage of them to choose their own goals, modes, and ways of work (Bateman and Crant, 1993). Furthermore, individuals high in proactive personality are active in realizing their objectives (Li et al., 2020). They transfer these opportunities into improving their working performance and make constructive changes. In addition, since both empowering leaders and proactive individuals recognize the importance of self-development, employees can obtain a higher degree of fit with their leaders and organizations. Extant studies have indicated that P-O fit can reduce role ambiguity and conflict (Verquer et al., 2003), which can result in a better perception of self-control toward their jobs and behaviors. Accordingly, the positive relationship between leaders’ empowerment and their followers’ autonomy needs is predicted to be higher for followers who are more proactive.

Alternatively, when employees are passive, the relationship may be mitigated. Individuals characterized by inactivity are reluctant to identify and utilize opportunities (Bateman and Crant, 1993). Thus, when they are confronted with opportunities given by their empowering leaders, they are not eager to identify and take advantage of them. Consequently, they lose the chance to improve their status quo, and further, their performance is estimated to be worse than that of their proactive counterparts. The positive influence of leaders’ empowering behavior on their competence needs may be weak due to the low satisfaction of their competence needs. Furthermore, passive individuals are not motivated to establish high-quality relationships with their leaders and colleagues because they adapt to their existing circumstances (Bateman and Crant, 1993). They do not closely communicate with their leaders regarding problems in their workplaces and receive feedback from their leaders (Li et al., 2010). Low-quality LMX can result in the dissatisfaction of the relatedness need since these employees fail to build connections with key figures in their external environments. Furthermore, although their leaders encourage teamwork, inactive individuals do not take the initiative to collaborate and communicate with other people. Compared to individuals who are characterized by proactivity, they are less capable of building social networks and thus have a lower degree of relatedness satisfaction. Third, passive employees may suffer from a low level of satisfaction of their autonomy needs. They conform to their status quo and lack the intention to make constructive changes (Bateman and Crant, 1993). When confronted with opportunities offered by leaders, they are inclined to present opposite patterns (Bateman and Crant, 1993). Either they are unable to identify opportunities or they give them up and do not make changes (Bateman and Crant, 1993). On this basis, we posit the following:

*H5*: Employees’ proactive personality positively moderates the positive relationship between leaders’ empowering behavior and employees’ self-determination such that the positive relationship between leader empowering behavior and employee self-determination is stronger for employees with high levels of proactive personality.

### The Moderated Mediation Model

As discussed above, it is predicted that leaders’ empowerment can positively influence their managed employees’ self-determination by satisfying their needs for autonomy, competence, and relatedness. This satisfaction then motivates knowledge-sharing behavior. Furthermore, the relationship between leaders’ empowering behavior and their followers’ self-determination is positively moderated by followers’ proactive personality, as individuals who are proactive can gain better satisfaction of their basic needs and subsequently enhance their feeling of self-determination. Moreover, enhanced self-determination pushes individuals to conduct more knowledge sharing with their colleagues. According to these statements, the indirect effect of leaders’ empowering behavior on employees’ knowledge sharing through employees’ self-determination is enhanced by employees’ proactivity. As such, we hypothesize the following:

*H6*: Employees’ proactive personality moderates the indirect effect of leader empowering behavior on employee knowledge sharing through employee self-determination such that the indirect effect is stronger when employees have high levels of proactive personality.

## Materials and Methods

### Participants

A three-wave longitudinal study with a 2-week time interval took place among employees in an R&D company located in Southwest China. We adopted a time-lagged study because temporal separation would help diminish common method variance (CMV) (Podsakoff et al., 2012). Questionnaires were distributed to all employees (N=400) in this company. To ensure the feasibility of our study, we performed a pilot test in advance by randomly selecting 98 samples to complete the questionnaire. The results indicated that the questionnaire had good reliability and validity. Therefore, it was possible to conduct a formal survey. At Time 1 (T1), 400 employees were invited to participate in the study (response rate 78%; *N*=312) to complete the questionnaire about leader empowering behavior and proactive personality. Two weeks later (T2), 400 employees were invited to participate again (response rate 71%; *N*=285) to measure self-determination. After another 2weeks (T3), 400 questionnaires were distributed to the employees (response rate 70%; *N*=278) to obtain data about knowledge-sharing behavior. The respondents were completely anonymous in the process of filling in the questionnaires and obtained certain material rewards upon completion. After matching data from T1, T2, and T3, 58% of the initial sample (*N*=230) was included in our empirical analysis. Among all the participants, 78.7% were female (*n*=181) and 21.3% were male (*n*=49). A total of 2.2% of the participants graduated from high school or below, 37.4% graduated from college, 53.5% had bachelor’s degree from universities, and 7% were master’s degree or above. Concerning job position, 83.9% were employee and 16.1% were first-line and middle managers. In addition, their average age was 38.90 (ranging from 22 to 58), average working tenure was 7.51years (ranging from 0.08 to 35.25), and average co-work duration with the current leader was 2.21years (ranging from 0.00 to 30.00).

### Measures

All measures used have been validated in previous research. Given that all original items were in English, we followed Brislin’s (1986) translation and back-translation procedures to ensure that all items were translated into Chinese properly. Each measure used a 6-point Likert-type scale ranging from “strongly disagree” to “strongly agree.”

#### Leader Empowering Behavior

To assess leader empowering behavior, we used Zhang and Bartol’s (2010) measure, which was adapted from Ahearne et al.’s (2005) measure. The 12-item measure has multi-item subscales corresponding to four dimensions: (1) enhancing the meaningfulness of work (three items, *α*=0.97, example item: “My manager helps me understand how my objectives and goals relate to that of the company”); (2) fostering participation in decision making (three items, *α*=0.92, example item: “My manager makes many decision together with me”); (3) expressing confidence in high performance (three items, *α*=0.93, example item: “My manager believes that I can handle demanding tasks”); and (4) providing autonomy from bureaucratic constraints (three items, *α*=0.92, example item: “My manager allows me to do my job my way”). A confirmatory factor analysis (CFA) for the 12-item scale indicated a single second-order factor solution with an acceptable fit [*χ*^2^ (50)=229.23, *p*<0.001; SRMR=0.05, IFI=0.95, CFI=0.95, RMSEA=0.13]. Cronbach’s alpha for the complete scale was 0.96.

#### Self-Determination

Self-determination was adapted from Broeck et al. (2010) Work-related Basic Need Satisfaction Scale. The 17-item scale has three dimensions: (1) NFA (six items, *α*=0.98, example item: “I feel free to express my ideas and opinions in this job”); (2) NFC (five items, *α*=0.94, example item: “I am good at the things I do in my job”); and (3) NFR (six items, *α*=0.77, example item: “At work, I feel part of a group/I do not truly feel connected with other people at my job”). The fit indexes for three first-order factors plus one second-order factor fell within an acceptable range [*χ*^2^(114)=347.09, *p*<0.001; SRMR=0.09, IFI=0.95, CFI=0.95, RMSEA=0.09], suggesting that the three dimensions reflected the construct. The overall Cronbach’s alpha was 0.91.

#### Proactive Personality

For proactive personality, the six-item scale (Cronbach *α*=0.94) was adapted from Bateman and Crant’s (1993) measure, which has been already used in previous studies (e.g., Parker et al., 2006; Li et al., 2010). Example item: “If I see something I do not like, I fix it.”

#### Knowledge-Sharing Behavior

Knowledge-sharing behavior was measured with Bock et al. (2005) five-item scale (Cronbach *α*=0.94). Example item: “I share my work reports and official documents with members of my organization.”

#### Control Variables

Six demographic variables of the followers’ gender (0 = male; 1 = female), age, education level (1 = high school or below, 2 = college, 3 = undergraduate, and 4 = master’s degree or above), position (1 = senior manager, 2 = middle manager, 3 = first-line manager, and 4 = employee), working tenure in the organization, and co-work time spent with leaders were included in the model as a set of control variables. Specifically, working tenure (in years) has been shown to have a negative relationship with knowledge sharing (Sarti, 2018). Then, we controlled for co-work duration with current leader, measured by the amount of time the individual employee has worked with his or her direct supervisor, which may exert an impact on the relationship between employee and supervisor. In addition, we took education level and position into account which may impact the ability and motivation of employees’ knowledge sharing. All of the control variables chosen here have been widely used in previous studies (i.e., Srivastava et al., 2006; Cheong et al., 2016; Nerstad et al., 2018; Stoermer et al., 2021).

## Results

### Confirmatory Factor Analysis

We conducted the CFA *via* Amos 23 to assess the discriminant validity of the measurement model. The results in Table 1 indicated that the hypothesized four-factor model fits the data well [*χ*^2^ (714)=1252.88, *p*<0.001, SRMR=0.07, IFI=0.95, CFI=0.95, RMSEA=0.06] and provides a significantly higher chi-square value than the alternative models. All the indicators loaded significantly on their corresponding latent second-order constructs. The results provided support for taking the four constructs as distinctive variables, and the four-factor model was thus retained for substantial hypothesis tests.

**Table 1:** Confirmatory factor analysis model fit results.

Models	*χ* ^2^	*df*	Δ*χ*^2^ (Δ*df*)	RMSEA	SRMR	CFI	IFI
**Four-factor model**							
The hypothesized four-factor model	1252.88	714	–	0.06	0.07	0.95	0.95
**Three-factor model**							
Combining KSB and self-determination	1447.15	717	194.27 (3)^***^	0.07	0.15	0.93	0.93
Combining self-determination and proactive personality	1472.61	717	219.73 (3)^***^	0.07	0.16	0.93	0.93
Combining KSB and proactive personality	2179.62	717	926.74 (3)^***^	0.09	0.16	0.87	0.87
**Two-factor model**							
Combining leader empowering behavior, self-determination, and KSB	2096.11	719	843.23 (5)^***^	0.09	0.21	0.87	0.87
Combining self-determination, proactive personality, and KSB	2371.53	719	1118.65 (5)^***^	0.10	0.18	0.85	0.85
**One-factor model**							
Combining all variables	3010.99	720	1758.11 (6)^***^	0.12	0.23	0.79	0.79

Δ*χ^2^ is derived from comparison with the hypothesized four-factor model. KSB, knowledge-sharing behavior. χ^2^, chi-square; df, degrees of freedom; RMSEA, root mean square error of approximation; SRMR, standardized root mean square residual; CFI, comparative fit index; and TLI, Tucker-Lewis index*.

*** *p*<0.001.

### Test of Common Method Bias

Due to the use of a single source of data, the potential impacts of common method bias should be examined. As shown in Table 1, the hypothesized four-factor model [*χ*^2^(714)=1252.88, SRMR=0.07, IFI=0.95, CFI=0.95, RMSEA=0.06] demonstrates better model fit indexes than the one-factor model [*χ*^2^(714)=3010.99, SRMR=0.23, IFI=0.79, CFI=0.79, RMSEA=0.12]. Next, we conducted Harman’s one-factor test (Podsakoff and Organ, 1986). The variance explained by the first factor from explanatory factor analysis is 30.10%, lower than the 50% threshold (Hair et al., 1998). In addition, the variance inflation factors for all variables are no more than 10. Thus, common method bias and multicollinearity issues are unlikely to have distorted the results of the present study.

### Hypothesis Test

Table 2 presents the descriptive statistics, correlations, and reliabilities of all the variables in our study. As expected, leader empowering behavior was significantly correlated with knowledge-sharing behavior (*r*=0.35, *p*<0.001) and self-determination (*r*=0.29, *p*<0.001). Self-determination was significantly correlated with knowledge-sharing behavior (*r*=0.27, *p*<0.001). Proactive personality was significantly correlated with leader empowering behavior (*r*=0.18, *p*<0.01) and positively related with self-determination (*r*=0.13, *p*>0.05) as well as knowledge-sharing behavior (*r*=0.06, *p*>0.05).

**Table 2:** Means, standard deviations, correlations, and reliabilities of studied variables.

S. No.		Mean	*SD*	1	2	3	4	5	6	7	8	9	10
1.	Age	38.90	8.38	–									
2.	Gender	0.79	0.41	0.18^*^	–								
3.	Position	3.80	0.48	−0.00	−0.10	–							
4.	Working tenure in the organization	7.51	9.22	0.35^**^	0.12	0.05	–						
5.	Co-work time spent with the leaders	2.21	3.77	0.26^**^	0.02	−0.06	0.21^*^	–					
6.	Education level	2.65	0.64	−0.52^**^	−0.10	−0.04	−0.11	−0.09	–				
7.	Leader empowering behavior	4.09	0.80	−0.21^*^	0.04	−0.18^*^	−0.08	0.00	0.14^*^	(0.96)			
8.	Self-determination	4.40	0.58	−0.15^*^	−0.01	−0.12	−0.14^*^	−0.14^*^	0.18^*^	0.29^**^	(0.91)		
9.	Proactive personality	3.81	0.83	−0.30^**^	−0.22^*^	−0.01	−0.10	−0.12	0.21^*^	0.18^*^	0.13	(0.94)	
10.	Knowledge-sharing behavior	4.52	0.65	0.02	0.13^*^	−0.16^*^	0.04	0.01	0.03	0.35^**^	0.27^**^	0.06	(0.94)

*N=230. Cronbach’s alphas are shown in parentheses along the diagonal. SD, standard deviation. Gender: 0 = male and 1 = female; Position: 1 = senior manager, 2 = middle manager, 3 = first-line manager, and 4 = employee; Education level: 1 = high school; 2 = technical college, 3 = undergraduate, and 4 = master and above; Age, working tenure, and co-work time spent with the leaders are in years*.

* *p*<0.05;

** *p*<0.01;

^***^p<0.001.

Hypothesis development was conducted using ordinary least squares regression and PROCESS Macro bootstrapping analysis in SPSS 26.0. All control variables (i.e., gender, age, education level, position, working tenure in the organization, and co-work time spent with the leaders) were included. The results are presented in Table 3. Model 5 revealed that hypothesis 1, which predicted that leader empowering behavior would be positively related to employees’ knowledge-sharing behavior, was found to be supported (*β*=0.28, *p*<0.001). Next, according to model 6, leader empowering behavior (*β*=0.24, *p*<0.001) and self-determination (*β*=0.23, *p*<0.01) were significantly related to employees’ knowledge-sharing behavior. Leader empowering behavior was positively related to self-determination (*β*=0.20, *p*<0.001) in model 2. Thus, it can be concluded that self-determination mediates the relationship between leader empowering behavior and knowledge-sharing behavior. One step further, we utilized PROCESS Macro Model 4 to test the mediation effect. The results showed that self-determination mediated the linkage from leader empowering behavior to knowledge-sharing behavior (*effect*=0.05, *SE*=0.03, *95% confidence interval* [*CI*]=[0.01, 0.10]). Therefore, Hypothesis 4 was supported, with self-determination acting as a partial mediator. Hypothesis 5 predicted that proactive personality would positively moderate the relationship between leader empowering behavior and employees’ self-determination. The results in model 3 showed that the latent interaction between leader empowering behavior and proactive personality was significantly related to self-determination (*β*=0.20, *p*<0.01). Thus, Hypothesis 5 received support.

**Table 3:** Ordinary least squares regression results.

Variables	Self-determination			Knowledge-sharing behavior		
	Model 1	Model 2	Model 3	Model 4	Model 5	Model 6
Intercept	4.39^**^ (4.65)	2.78^*^ (2.82)	2.31^*^ (2.30)	4.84^**^ (1.09)	2.56^*^ (1.11)	1.93 (1.11)
**Controls**						
Age	0.04 (0.19)	0.21 (0.98)	0.34 (1.57)	0.05 (0.25)	0.29 (0.24)	0.24 (0.24)
Working tenure in the organization	−0.03 (−1.31)	−0.03 (−1.32)	−0.04 (−1.70)	0.01 (0.03)	0.01 (0.03)	0.02 (0.03)
Co-work time spent with the leaders	−0.06 (−1.68)	−0.06 (−1.96)	−0.06 (−1.96)	−0.01 (0.04)	−0.02 (0.04)	−0.00 (0.04)
Education level	0.15^*^ (2.19)	0.15 (2.21)	0.14^*^ (2.11)	0.04 (0.08)	0.04 (0.08)	0.00 (0.08)
Gender	0.05 (0.50)	−0.03 (0.27)	0.04 (0.43)	0.18 (0.11)	0.14 (0.10)	0.14 (0.10)
Position	−0.14 (−1.80)	−0.09 (−1.13)	−0.11 (−1.45)	−0.20^*^ (0.09)	−0.12 (0.09)	−0.10 (0.09)
**Independent variable**						
Leader empowering behavior		0.20^**^(4.22)	0.19^**^ (4.15)		0.28^**^ (0.05)	0.24^**^ (0.05)
**Mediator**						
Self-determination						0.23^*^ (0.08)
**Moderator**						
Proactive personality			0.03 (0.65)			
**Two-way interaction**						
Leader empowering behavior *Proactive personality			0.20^*^ (3.42)			
*R* ^2^	0.07	0.14	0.19	0.04	0.15	0.19
*Adjusted R* ^2^	0.05	0.07	0.15	0.01	0.12	0.16
Δ *Adjusted R*^2^		0.02	0.10	-	0.11	0.15
*F*	2.79^*^	5.12^**^	5.50^**^	1.50	5.51^**^	6.18^**^

*N=230. Statistics reported are unstandardized regression coefficients (and standard errors); LLCI, lower-level confidence interval tested at 95% significance level; and ULCI, upper-level confidence interval tested at 95% significance level*.

* *p<0.05*;

^*^p<0.01;

** *p<0.001*.

**Figure 2:**
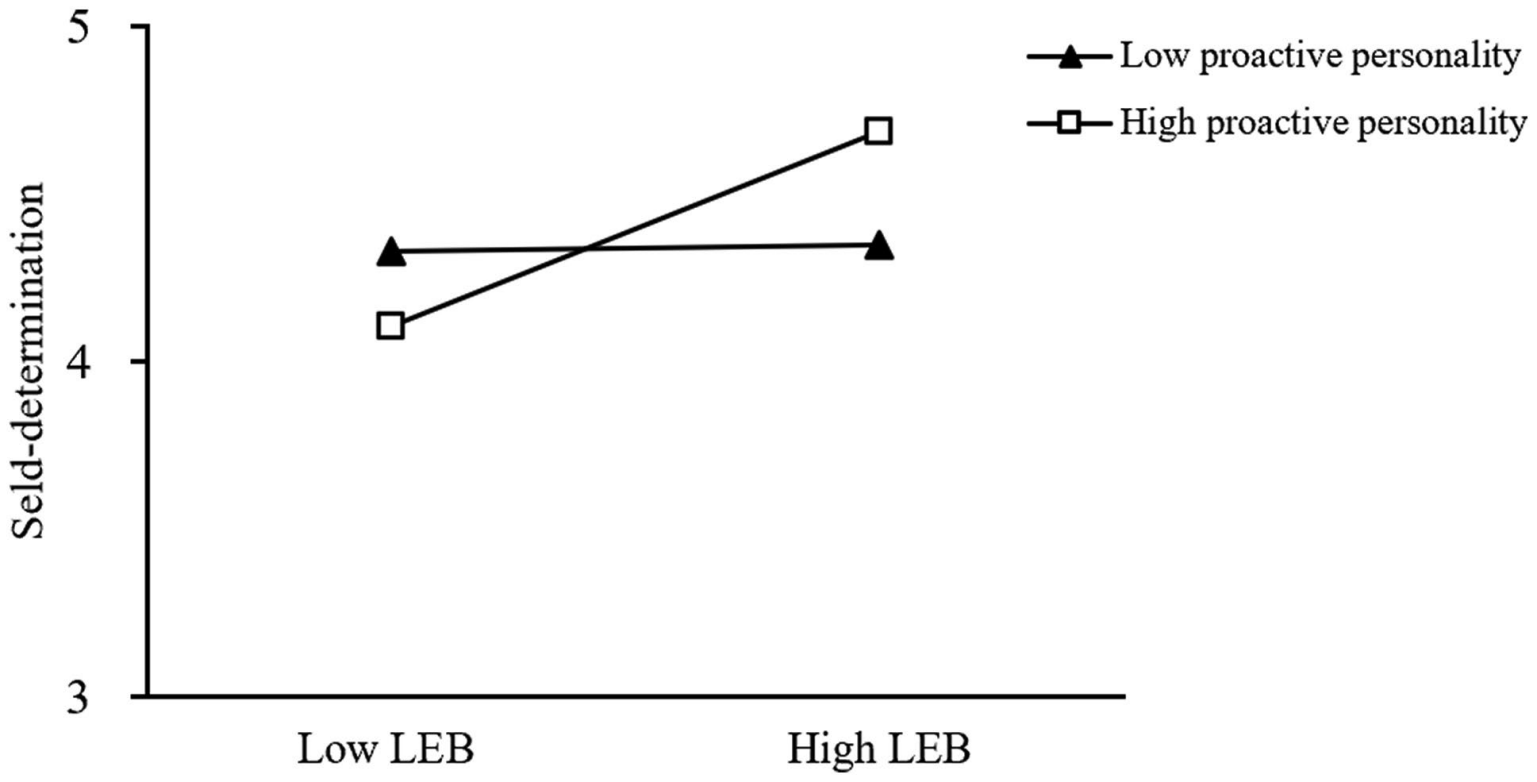
Simple Slope of Proactive Personality. LEB means leader empowering behavior.

We used the procedure outlined by Aiken and West (1991) to plot high and low levels of the moderator. Figure 2 depicts the pattern of the moderated results. A simple slope test showed that the extent to which leader empowering behavior was related to employees’ self-determination depends on the level of proactive personality. Specifically, when proactive personality was high (one standard deviation above the mean), leader empowering behavior had a stronger relationship with employees’ self-determination (*simple slope*=0.36, *effect*=0.02, *SE*=0.07, *t*=5.37, *p*<0.001) than it did under the low level of proactive personality (one standard deviation below the mean; *simple slope*=0.02, *effect*=0.36, *SE*=0.07, *t*=0.32, *p*>0.1), suggesting that the effect of leader empowering behavior on employees’ self-determination achieve the highest level when employees are equipped with high-level proactive personality. Therefore, Hypothesis 5 was partially supported, suggesting that employees’ high-level proactive personality positively moderated the relationship between leader empowering behavior and self-determination.

As a robustness check, following Preacher et al. (2007) recommendations, we utilized the PROCESS Macro for SPSS to test the complete moderated mediation model *via* 5,000 bootstrap resamples to construct 95% bias-corrected confidence intervals. The results in Table 4 showed that the moderated mediation is signification (*effect*=0.05, *SE*=0.03, *95%* CI=[0.00, 0.11]), suggesting that employees’ proactive personality positively moderated the relationship between leader empowering behavior and self-determination, which further exerted an influence on employees’ knowledge-sharing behavior. Thus, Hypothesis 6 received support.

**Table 4:** Moderated mediation results of bootstrapping method with 5,000 resamples.

Conditional effects of leader empowering behavior on self-determination					
Condition	Leader empowering behavior	Effect	*SE*	Boot 95% CI	
				LLCI	ULCI
Low (M−1 *SD*)	3.28	0.01	0.02	−0.02	0.05
Mean		4.10	0.04	0.02	0.01	0.10
High (*M*+1 *SD*)	4.89		0.08	0.04	0.01	0.18
**Index of moderated mediation**					
Leader empowering leadership→knowledge-sharing behavior (*via* self-determination)		Effect	*SE*	LLCI	ULCI
		0.05	0.03	0.00	0.11

*SE, standard errors; LLCI, lower-level confidence interval tested at 95% significance level; and ULCI, upper-level confidence interval tested at 95% significance level*.

## Conclusion and Contribution

### Conclusion

Drawing on SDT, this paper aimed to test the underlying mechanism and boundary conditions on the influence of leader empowering behavior on employee knowledge sharing. Based on a time-lagged survey designed for 230 employees, the empirical findings revealed that leader empowering behavior was positively related to employee knowledge sharing; the relationship between leader empowering behavior and employee knowledge sharing was mediated by employees’ self-determination; and proactive personality positively moderated the relationship between leader empowering behavior and employee self-determination and further affected employee knowledge sharing through the mediating role of employee self-determination.

### Theoretical Contribution

Our study makes several contributions. First, by examining the positive influence of leader empowering behavior on employee self-determination, our study contributes to the empowering leadership literature. Previous studies have demonstrated that leader empowering behavior can be considered as leader supportive behavior, which commonly brings positive results, including improving organizational performance (Carmeli et al., 2011), team performance (Srivastava et al., 2006; Tung and Chang, 2011; Lorinkova et al., 2013), individual self-efficacy (Conger and Kanungo, 1988; Arnold et al., 2000), innovation behavior (Zhang and Bartol, 2010), and individual performance (Harris et al., 2014; Humborstad et al., 2014). We found that leader empowering behavior promotes the satisfaction of individuals’ basic psychological needs and thus enhances their self-determination. This is in line with previous studies and supports the positive impact of leader empowering behavior. Moreover, to our knowledge, our study is the first attempt to explore how leader empowering behavior influences employees’ self-determination. This enriches the literature on leader empowering behavior by introducing SDT.

Second, we contribute to the existing literature by exploring the underlying mechanism of the relationship between leader empowering behavior and employee knowledge sharing from the perspective of SDT. We found that leader empowering behavior promoted employee knowledge sharing through the indirect effect of employee self-determination. This empirically verifies Gagné’s (2009) model of knowledge-sharing motivation, in which self-determination is a critical factor for knowledge sharing. Furthermore, our finding enriches this model by emphasizing the role of leaders in affecting employees’ self-determination. In addition, scholars have commonly emphasized the mediating mechanism of psychological empowerment (Zhang and Bartol, 2010; Dewettink and Ameijde, 2011) and attitude (Xue et al., 2011) in the relationship between leader empowering behavior and employee knowledge sharing. This paper shows that self-determination also plays a mediating role in this relationship. This provides a new perspective for the research of leader empowering behavior and knowledge sharing.

Finally, this paper also explores the boundary conditions in this mechanism. Self-determination theory explains work motivation and indicates that there exist various degrees of behavioral initiation and regulation in workplaces (Gagné and Deci, 2005). Accordingly, employees’ psychological mechanisms and reactions differ in response to their supervisors’ empowerment. As an aspect of positive psychology, a proactive personality has been proven to strengthen the positive influence of leaders’ empowering behavior on their followers’ knowledge sharing. This is in line with previous findings that have investigated the positive effects of proactivity (e.g., Bateman and Crant, 1993; Fuller and Marler, 2009; Li et al., 2010). In addition, we included proactive personality as a moderating variable and proved its synergy with empowerment. Our findings suggest that the effect of leaders’ empowering behavior on employees’ self-determination is higher when individuals are proactive owing to the increased satisfaction of their autonomy, competence, and relatedness needs. Furthermore, the influence is transferred to increase their knowledge-sharing behavior.

### Practical Contribution

On the one hand, leaders can promote employees’ knowledge sharing through empowering behavior. This paper shows that leader empowering behavior has a significant positive impact on employees’ knowledge sharing. Therefore, managers can enhance opportunities, intentions, and motivations for knowledge sharing among subordinates through empowerment practices. For instance, leaders could promote the transformation of the organizational structure from a traditional management structure to empowered teamwork. In such a way, leaders could provide more empowerment practices, such as encouraging opportunity thinking and self-development, which gives employees the ability to share knowledge with others. They can encourage employee self-reward, participative goal setting, and independent action, which motivates employees to share knowledge with others. In addition, they can encourage teamwork and participatory decision making, which gives employees the opportunity to share knowledge with others.

On the other hand, leaders should help employees foster self-determination and proactive personality. Our study shows that self-determination can promote employees’ knowledge sharing. Therefore, to facilitate employees’ knowledge sharing, it is necessary for leaders to meet employees’ needs of autonomy, competence, and relatedness by providing appropriate managerial practices. For example, providing more self-discretion could satisfy employees’ needs for autonomy, training employees with necessary occupational skills could satisfy employees’ needs for competence, and encouraging more team work could help satisfy employees’ needs for relatedness. In addition, our study also reveals that when empowered by their leaders, individuals with high levels of proactive personality would feel more self-determined and be more likely to share knowledge than their peers. Therefore, leaders could encourage employees to be more proactive, such as to be more initiative, practice identifying, taking advantage of opportunities, and seek information and opportunities.

### Limitations and Future Directions

This study has achieved certain results. Despite its contributions to theory and practice, we note some shortcomings, which are worth further research and improvement in the future. Specific research limitations and future research directions are as follows:

First, this paper adopts a three-wave study design with a 2-week time lag between leaders’ empowering, employees’ self-determination, and knowledge-sharing behavior. We decided to use the time delay because it may help diminish CMV as a kind of temporal separation (Podsakoff et al., 2012). In addition, this design outperforms the cross-sectional design because it can examine causation over time (Tims et al., 2016). By collecting the variables in sequence with a time lag, we can prove the causal effect between leaders’ empowerment, employees’ self-determination, and knowledge sharing more effectively. Based on our regression results, the correlations between the three variables are stable over the period. This verifies our assumptions of the directions of the relations between leaders’ empowering behavior, their managed staff’s self-determination, and knowledge-sharing behavior.

Nevertheless, there are drawbacks of the research design. First, the three-wave design entails added difficulty in data collection. More importantly, we employed self-reports, as all the variables were scored by employees. Although it passed the homogeneity test, CMV was still possible. The regression results may suffer from overevaluation of the true correlations (Podsakoff et al., 2012). Therefore, in the future, researchers can supplement our results by obtaining measures from different sources (Podsakoff et al., 2012). Future research can measure different data sources, such as evaluating each member’s knowledge-sharing behavior within the team and measuring the organization’s job characteristics in the human resources department. By doing so, the results of this paper can be further verified to obtain a better understanding.

In addition, this paper adopts a questionnaire survey to obtain data and test the research hypotheses. However, the questionnaire survey method can verify only the correlation between the research variables. To better test the possible causal relationship in the hypothesis model, future research should apply experimental methods to make further verifications. For example, the experimental group and the control group should be set up to study whether the experimental group will affect individual knowledge sharing when leader empowering behavior is added. Furthermore, although the data collected in the context of China supported our theoretical model, future studies are encouraged to extend our model in different contexts to generalize the findings.

There are other findings in this study worth future research. Our empirical findings show that in context of low leader empowering behavior, individuals with a low proactive personality exhibit higher self-determination than their colleagues with a highly proactive personality. This phenomenon may be explained by trait activation theory, which argues that personal traits are activated with trait-relevant situational cues (Tett and Burnett, 2003). Moreover, low leader empowering behavior is a strong situation, as leaders provide clear guidelines and direct orders to employees rather than providing unsupervised freedom (Judge and Zapata, 2015). From this theoretical perspective, under low leader empowering behavior with strict constraints, proactivity seems to be inappropriate given the high risks of proactive actions. Consequently, the trait-irrelevant context cannot enable proactive employees to stimulate their personality traits and take advantage of them.

## Data Availability Statement

The raw data supporting the conclusions of this article will be made available by the authors, without undue reservation.

## Ethics Statement

The studies involving human participants were reviewed and approved by School of International Business, Southwestern University of Finance and Economics. Written informed consent for participation was not required for this study in accordance with the national legislation and the institutional requirements.

## Author Contributions

SX mainly led research design, literature review, hypotheses developing, data analysis, and paper drafting. YZ was mainly responsible for literature review and hypotheses developing. NN mainly led literature review, data collection, and paper proofreading. SW was mainly responsible for data collection and data analysis. WC was mainly responsible for data collection and proofreading. All authors contributed to the article and approved the submitted version.

## Conflict of Interest

The authors declare that the research was conducted in the absence of any commercial or financial relationships that could be construed as a potential conflict of interest.

## Publisher’s Note

All claims expressed in this article are solely those of the authors and do not necessarily represent those of their affiliated organizations, or those of the publisher, the editors and the reviewers. Any product that may be evaluated in this article, or claim that may be made by its manufacturer, is not guaranteed or endorsed by the publisher.
